# Seasonal and Geographic Distribution of Cercarial Infection in *Lymnaea gedrosiana* (Pulmunata: Lymnaeidae) In North West Iran

**Published:** 2013

**Authors:** Abbas IMANI-BARAN, Mohammad YAKHCHALI, Reza MALEKZADEH-VIAYEH, Ali FARAHNAK

**Affiliations:** 1 Dept. of Pathobiology, Parasitology Division, Faculty of Veterinary Medicine, Tabriz University, Tabriz, Iran; 2 Dept. of Pathobiology, Parasitology Division, Faculty of Veterinary Medicine, Nazlu campus, Urmia University, Urmia, Iran; 3 Artemia and Aquatic Animals Research Institute, Urmia University, Urmia, Iran; 4 Dept. of Parasitology and Mycology, School of Public Health, Medical Sciences of Tehran University, Tehran, Iran

**Keywords:** Cercariae, *Lymnaea gedrosiana*, Iran

## Abstract

**Background:**

Trematodes are a diverse group of endoparasites which require molluscan and vertebrate animals as intermediate and definitive hosts in their life cycle. The present study was carried out to determine the diversity and geographic distribution of infection with trematodes'cercariae in the snail *Lymnaea gedrosiana* from north-west Iran.

**Methods:**

A total number of 6759 Lymnaeidae snails were collected from 28 snail habitats; of these *L. gedrosiana* was the prevalent snail (74.37%) which examined for cercarial infection by shedding method.

**Results:**

The overall infection rate was 8.03%. The most frequent trematodes cercariae in the snail were xiphidiocercariae (81.98%), furcocercariae (32.26%), echinostome cercariae (5.19%), and monostome cercariae (1.24%). The highest infection rate in *L. gedrosiana* (100%) was with echinostome cercariae from Golestaneh in autumn.

**Conclusion:**

Due to the important role of pond snails in transmission of cercariae to fish as a source of zoonotic diseases, it is essential to estimate the distribution and abundance of the snails and the rate of their infection with different trematodes’ cercariae, and establish control programs in each region.

## Introduction

Digenian trematodes have complicated life cycles in which molluscs play the key role as intermediate hosts for part of their developmental stages. In this regard, freshwater snails, in particular those from the order Basomamtophora, have substantial contribution to development and transmission of parasitic flukes. For instance, some 20 species of cercariae have been isolated from the lymnaeid snails of *Lymnaea peregra* (Muller, 1774) (1). Snail-mediated diseases are among the major groups of helminthic diseases caused by trematode parasites. However, the main snail species involving in the transmission of flukes vary in different geographical regions.

Freshwater snails have been studied in different Iranian provinces including Fars, Khoozestan and Mazandaran (2–5). *L. gedrosiana* (Annandale and Prashad, 1919) has been reported to be a prefered intermediate host for a number of parasitic helminths such as *Fasciola gigantica* (Cobbold, 1855) (6), *Ornitobilharzia turkestanicum* (Skrjabin, 1913) (2, 7), and *Trichobilharzia* spp. (3). It was also found that *L. gedrosiana* had a considerable role in the transmission of zoonotic diseases such as cercarial dermatitis (1.1% in South-West and 0.05% in North of Iran), fasciolosis (0.35%), Plagiorchids infections (0.1%), and *Clinostomum* infections (0.2%) in Iran (3, 6, 8–10). Therefore, examination of the snails makes it possible to gain information about the degree to which they are responsible for infection distribution. This is also the keystone for identifying the trematode fauna in the areas of interest.

To date, no large-scale study has been carried out on the distribution and abundance of *L. gedrosiana* and its contribution to the transmition of cercarial infection in north-western Iran. Thus, the aim of this study was to elucidate the seasonal and regional incidence of *L. gedrosiana* and its rate of cercarial infections in the region.

## Material and Methods

### Study area

**Fig. 1:**
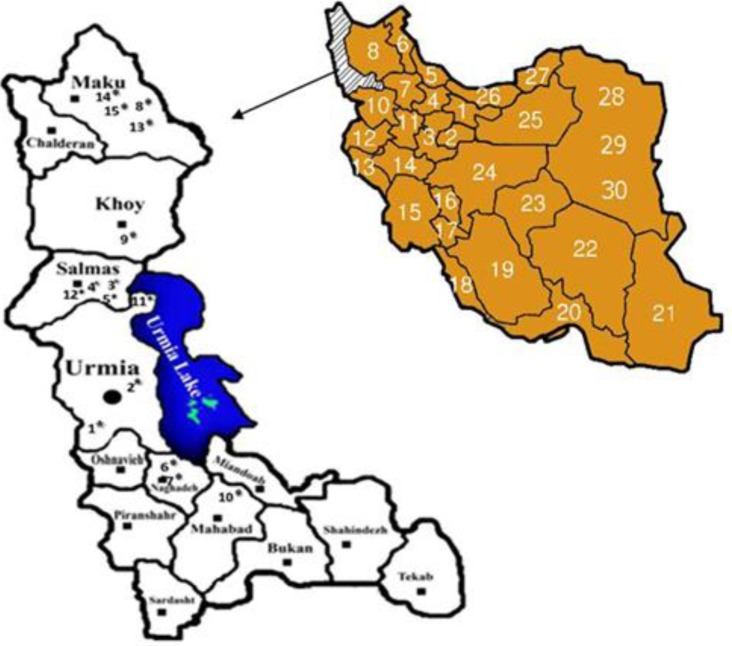
Map of the collected *Lymnaea gedrosiana* with cercariae infection in West Azerbaijan province, Iran (1. Ziveh, 2. Osaloo, 3. Najafabad, 4. Esmjondi, 5. Urmia-Goushji Road, 6. Shorgul, 7. Eslamabad, 8. Gargulug, 9. Shabanlu, 10. Darlak, 11. Gugarchingaleh, 12. Garehbagh, 13. Garehaghaj, 14. Esmailkandi, 15. Marganlar)

West Azerbaijan Province is located in north-west of Iran (35°46′ to 39°58′ in latitude and 44°3′ to 47°23′ in longitude) (Fig. 1). Excluding the Lake Urmia, this semi-humid and temperate province has an area of 37,608 km^2^ elevating 1,332m above sea level. The climate of the province is largely influenced by the rainy winds of the Atlantic Ocean and Mediterranean Sea; the maximum temperature reaches 34°C in July, while minimum temperature may be –16°C in January. Annual precipitation varies between 300 and 800 mm with large yearly and monthly fluctuations. Generally, the province witnesses two rainy seasons, the first from March to May and the second in October-November (5). Three are numerous water bodies and reservoirs with relatively appropriate environmental conditions in West Azarbaijan province where suitable habitats are provided for pond snails (5).

### Snail collection

A total of 28 perennial and seasonal freshwater snails habitats were monitored from May to December 2010 (Fig.1). The habitats included various water-body types, i.e. wetlands, ponds, rivers, canals, springs, swamps, pools, streams and ditches, located in both mountainous and low-land areas of north, central and south parts of the province. Snail sampling was undertaken by searching each site for 15 minutes using a standard flat wire mesh scoop with a mesh size of 2mm (11, 12). The collected snails were placed in plastic screw cap containers containing the water of snail habitat and transferred alive to the laboratory for species identification using the morphological keys provided by Mansoorian (13) and Pfleger (1). The identified snails were kept alive in an aquarium at optimal conditions to be investigated for their cercarial infection.

### Collection of cercariae from the infected snails

The identified snails as *L. gedrosiana* were transferred to the Parasitology Museum of the Tehran Faculty of Veterinary Medicine for detailed characterization and verification. The snails were then placed individually in flat-bottomed glass vials (height 7.5 cm, diameter 2.5 cm) containing filtered pond water and exposed to a 100-W light bulb at a distance of 15cm for 4-6 hours for cercarial shedding (14). The snails which did not shed cercariae on the first exposure were re-exposed on the second day. Cercariae were characterized by morphological and biometrical examinations as described by Frandsen and Christensen (15).

### Statistical evaluation

Data were analyzed by SPSS statistical program (version 14, SPSS Inc., Chicago, IL, USA) using the non-parametric Chi-square test with confidence interval of 95%. Probability of < 0.05 was regarded as significant.

## Results

### Snails

Of the total of 6759 collected Lymnaeidae snails, *L. gedrosiana* was the predominant specie (74.37%) observed in 18 out of the 28 investigated water bodies (Fig. 1). The snail was found mainly in the stagnant or slow-moving, clear to slightly turbid waters with aquatic plants cover. During the course of this study, the seasonal and geographical distributions of *L. gedrosiana* were significantly different (*P* = 0.0001). However, there was no significant differences in the distributions for the snail in Ziveh (*P* = 0.816) and Gogarchinghaleh (*P* = 0.677). The snails counts were significantly higher in summer than in autumn (*P* = 0.0001) (Table 1).

**Table 1:** Association between regional and seasonal distribution of *Lymnaea gedrosiana* population in northwestern Iran (n = 5026)

Place	Season		*P*
	Summer	Autumn	
**Ziveh**	38	36	0.816
**Najafabad**	181	0	0.0001
**Shorgol**	156	121	0.035
**Ghargologh**	30	15	0.025
**Shabanlu**	222	154	0.0001
**Gogarchinghaleh**	188	180	0.677
**Gharahaghaj**	344	406	0.024
**Esmailkandy**	372	175	0.0001
**Marganlar**	294	0	0.0001

### Diversity and abundance of cercariae

From the 3673 identified *L. gedrosiana* snails, 8.03% were infected with cercariae of different trematodes (Table 2). The infections were observed throughout the study period, but the largest number of the infected snails was observed between June and August (Table 3). Identified cercariae and their respective contribution to the total snail infection rate were as follows: xiphidiocercariae 81.98%, furcocercariae 32.26%, echinostome cercariae 5.19%, and monostome cercariae 1.24% (Table 2). Xiphidiocercariae and monostome cercariae were found only in the snails sampled from north part of West Azarbaijan province, while echinostome cercariae and furcocercariae were absent from the snails sampled in the central part of the province. All examined snails (100%) from Golestaneh in autumn were infected with echinostome cercariae, and the snails sampled from Gharahaghaj in summer had the highest infection rate with xiphidiocercariae (76.81%) (Table 3).

**Table 2:** Geographical distribution of cercariae infection in examined *Lymnaea gedrosiana* snails of northwestern Iran (n = 3673)

Ccercariae	Place	Snail	
		No. of examined snails	Prevalence (%)
**Xiphidiocercariae**	Shorgul	277	2.17
	Gharahaghaj	750	81.98
	Marganlar	294	10.2
	Gharahbaagh (Zanoil)	278	2.88
	Gharahbaagh (Jamgoli)	242	0.83
**Furcocercariae**			
	Marganlar	294	5.44
	Gargulug	30	10.02
	Shorgul	277	3.61
**Echinostome cercariae**	Zarineh-roud	31	32.26
	Golestaneh	318	5.19
	Shorgul	277	3.61
	Marganlar	294	3.43
	Darlak	60	1.67
**Monostome cercariae**	Gargulug	30	1.24
	Gharahbaagh (Kefi)	261	1.15
**Total**	-	3673	8.03

**Table 3:** Seasonal distribution of cercarial infection in examined *Lymnaea gedrosiana* snails of northwestern Iran (n = 403)

Ccercariae	Place	Season	Snail	
			No. of examined snails	Prevalence (%)
**Xiphidiocercariae**	Shorgul	Summer	156	3.85
	Gharahaghaj	Summer	344	76.81
	Gharahaghaj	Autumn	406	5.17
	Marganlar	Summer	294	10.24
	Gharahbaagh (Zanoil)	Autumn	278	2.88
	Gharahbaagh (Jamgoli)	Autumn	242	0.83
**Furcocercariae**	Shorgul	Summer	156	4.49
	Shorgul	Autumn	121	2.48
	Marganlar	Summer	294	5.44
	Gargulug	Summer	30	10.02
**Echinostome cercariae**	Zarineh-roud	Summer	31	32.26
	Golestaneh	Summer	75	30.6
	Golestaneh	Autumn	243	100
	Shorgul	Summer	156	3.85
	Shorgul	Autumn	121	3.31
	Marganlar	Summer	294	3.44
	Darlak	Summer	60	1.67
**Monostome cercariae**	Gargulug	Summer	30	6.67
	Gharahbaagh (Kefi)	Autumn	261	1.15

## Discussion

Lymnaeidae snails are of medical and veterinary importance since they are required, as intermediate hosts, to complete the life cycle of trematode species. They are distributed throughout the world and are known as the vectors of more than 71 species belonging to 13 trematode families (16). A considerable body of research has explored the potential role of lymnaeid snails in transmitting the infectious parasitic trematodes worldwide (17–19). However, intra-molluscan trematode parasitism is frequently associated with the alteration of a host's growth, fecundity or survival (20), and its susceptibility to trematodes (21). In the present study, *L. gedrosiana* was found to be a predominant pond snail in the region. This finding was in accordance with previous reports from Iran (4, 5, 13, 22, 23). Furthermore, in consitence with several earlier studies (2, 4, 13, 24), in this study the highest population density of *L. gedrosiana* was recorded in summer.

Until present, only a few studies have been carried out on the diversity and abundance of cercarial infection in the pond snails of Iran. For instance, cercarial infection in *L. gedrosiana* was reported from Khoozestan province (3, 25), in *Galba truncatula* (Müller, 1774) from Khoozestan and Kurdestan provinces (26), and in *L. gedrosiana* and *L. palustris* (Müller, 1774) from northern Iran (6, 9, 10). The snail *L. gedrosiana* is found to be a general intermediate host for four groups of cercariae in the studied region. Several studies have confirmed the simultaneous infection of *L. gedrosiana* with echinostome cercariae (Echinostomatidae), furcocercariae (*O. tur-kestanikum* and *Trichobilharzia* spp.), monostome cercariae (Notocotylidae), and xip-hidocercariae (Plagiorchiidae) (2, 3, 24) in Iran. Sharif et al. (10) found that *L. gedrosiana* in northern Iran were also infected with the same cercariae types. Loy and Haas (27) isolated the larvae of 18 trematode species from *L. stagnalis* in Germany. Faltynkova et al. (18) identified 24 trematode species comprising 19 cercariae in *L. stagnalis*, of which the dominant cercariae were those belonging to three species of *Echinoparyphium aconiatum*, *Opisthioglyphe ranae*, and *Plagiorchis elegans*. Immani-Baran et al. (28) found the infection of *L. auricularia* snails in North West Iran with two groups of fluke's cercariae, i.e. furcocercariae and echinostomcercariae.

Seasonality that is mirrored by changes in environmental variables can intervene in snail's ecology and influence the larval development of a trematode inside its host snail. It may also affect cercarial shedding (the release of cercariae from the host snail in nature). However, the influence of environmental elements on cercarial shedding is trematode-specific (29). The optimal reproduction of *L. gedrosiana* in northwestern Iran occurs in early summer (June-July) (2, 4). Similarly, the highest cercariael infection rates in lymnaeid snails of the region were observed between June and September, while Sharif et al. (10) recorded the maximum infection rates in late summer (August-September). Thus, it can be anticipated that both snail's propagation and their infection with trematodes are correlated with seasonal variations. Farahnak et al. (30) noted that various ecological factors such as season and water temperature, pH and dissolved oxygen influence the emergence of cercariae from the snails and their release inside the water resources.

## Conclusion

With regard to the importance of farm animal health in national economy, it is essential to study the diversity, distribution and abundance of the intermediate hosts of infectious trematodes, mainly freshwater snails. *L. gedrosiana* is a common pond snail in West Azarbaijan province which has shown the capacity for vectoring diverse cercarial species. Results of this study and those of the related investigations can assist in collecting data on the ecological relevance of the snails distribution and the pattern of transmission of digenian trematodes by the snails and finally, in prevention and control of the following disease outbreaks.
